# Chemical Characteristics and Antimicrobial Activity of *Arctostaphylos uva-ursi* (L.) Spreng. Extracts Against Skin-Associated Bacteria

**DOI:** 10.3390/molecules31081267

**Published:** 2026-04-12

**Authors:** Danuta Sugier, Aleksandra Nurzyńska, Małgorzata Miazga-Karska, Łukasz Sęczyk, Piotr Sugier

**Affiliations:** 1 Department of Industrial and Medicinal Plants, University of Life Sciences in Lublin, 15 Akademicka Street, 20-950 Lublin, Poland; danuta.sugier@up.lublin.pl (D.S.); lukasz.seczyk@up.lublin.pl (Ł.S.); 2 Chair and Department of Biochemistry and Biotechnology, Medical University of Lublin, 1 Chodźki Street, 20-093 Lublin, Poland; malgorzata.miazga-karska@umlub.edu.pl; 3 Department of Botany, Mycology and Ecology, Institute of Biological Sciences, Maria Curie-Skłodowska University, 19 Akademicka Street, 20-033 Lublin, Poland

**Keywords:** *Arctostaphylos uva-ursi*, *Cutibacterium acnes*, bearberry extracts, antioxidant activity, cytotoxicity, antibacterial activity

## Abstract

The interest in the use of phytochemicals and herbal medicines for the treatment of acne vulgaris has grown steadily over recent decades. The research on the secondary metabolites and biological properties of bearberry (*Arctostaphylos uva-ursi* (L.) Spreng.) has been intensified in recent years, but the range of bacterial strains tested, many of which are highly relevant to human health, remains very limited. Therefore, the aim of this study was to evaluate the chemical composition and the antioxidant, antimicrobial, and cytotoxic activities of water and ethanolic bearberry leaf extracts. Compared with the ethanolic extract, the water extract was characterized by higher concentrations of arbutin, hydroquinone, corilagin, and hyperoside and the absence of ursolic acid and oleanolic acid. However, it exhibited lower total phenolic content and reduced levels of penta-O-galloyl-β-d-glucose (PGG). The ethanolic extract of bearberry leaves showed higher antioxidant activity and the most favorable overall biological properties. The therapeutic index (TI) values for the water and ethanolic extracts, respectively, were as follows: *Cutibacterium acnes* ATCC 11827 (10.70; 21.57), *Propionibacterium acnes* PCM 2334 (10.70; 43.13), *P. acnes* PCM (5.33; 21.57), *Staphylococcus aureus* ATCC 25923 (10.70; 21.57), and *S. epidermidis* ATCC 12228 (5.33; 10.78). The present findings further support the medicinal and cosmetic use of *A. uva-ursi* and highlight its potential as a source of natural antibacterial agents for acne treatment.

## 1. Introduction

*Arctostaphylos uva-ursi* (bearberry) is a medicinal plant with considerable ethnopharmacological relevance and a chemically diverse phytocomplex. Its leaves (*Uvae ursi folium*) have been traditionally used, particularly in European herbal medicine, to manage lower urinary tract infections (UTIs). Historical records also describe their use in the treatment of heartburn, nephrolithiasis, cystolithiasis, fluid retention, and hyperglycemia [1,2,3,4]. Based on the longstanding traditional use and accumulated pharmacological evidence, European regulatory authorities, including the European Medicines Agency, have recognized bearberry leaf preparations as adjuvants for the symptomatic relief of mild urinary discomfort, such as frequent and painful urination [5,6,7,8,9].

The therapeutic application of bearberry in urinary disorders has primarily been attributed to its antimicrobial, anti-inflammatory, and mild diuretic properties. However, emerging evidence indicates a broader spectrum of biological activities associated with its phytoconstituents, including antioxidant, antiproliferative, hypoglycemic, hepatoprotective, and neuroprotective effects [5,10,11]. In vitro and in vivo preclinical studies have demonstrated its free radical-scavenging capacity, inhibition of abnormal cell proliferation, modulation of glucose metabolism, hepatocellular protection, and neuroprotective potential. These findings have expanded the scientific interest in bearberry beyond its conventional use in phytotherapy [12,13].

The principal bioactive compound in bearberry leaves is arbutin, a hydroquinone glycoside largely responsible for its antimicrobial activity in the urinary tract. When combined with conventional chemotherapeutic agents, arbutin-containing extracts enhance therapeutic efficacy and reduce toxicity in the treatment of UTIs, compared with the use of chemotherapeutic agents alone [14]. In addition to arbutin, bearberry leaves contain derivative constituents such as methylarbutin and free hydroquinone, and a wide array of other secondary metabolites [10,12,15], including phenolic glycosides (e.g., picein), galloylated glucose derivatives, flavonoids, condensed tannins, triterpenes, organic acids, and vitamins [6,9,10]. Collectively, this complex phytochemical composition and pharmacological profile of *A. uva-ursi* underscore its continued relevance as a medicinal plant and warrant further investigation into its therapeutic applications and mechanisms of action.

Acne vulgaris, a chronic inflammatory disorder of the pilosebaceous unit, presents with non-inflammatory (comedones) and inflammatory lesions (papules, pustules, and nodules). Its multifactorial pathogenesis involves follicular hyperkeratinization, increased sebum production, microbial dysbiosis, and complex immune responses [16,17,18,19,20]. A key contributor is *Cutibacterium acnes*, an anaerobic bacterium that constitutes a major component of the normal skin microbiota [18,21]. The bacterium triggers innate immunity by activating Toll-like receptors 2 and 4 (TLR2/4) on keratinocytes and monocytes, inducing the release of pro-inflammatory cytokines like IL-1α, IL-8, and TNF-α. This cascade recruits neutrophils, amplifies local inflammation, and forms lesions. Additionally, *C. acnes* produces lipases that oxidize sebum lipids, specifically squalene, thereby activating NF-κB and enhancing inflammation [22,23].

Increasing antibiotic resistance in *C. acnes* strains represents a growing clinical challenge, limiting the long-term effectiveness of conventional antimicrobial therapies and necessitating the development of alternative or adjunctive approaches [19]. Antioxidants have emerged as promising candidates for acne management, as they neutralize reactive oxygen species (ROS), thereby reducing oxidative stress and attenuating downstream inflammatory pathways [24]. Many natural antioxidants also exhibit beneficial activities, including anti-inflammatory and antimicrobial effects against *C. acnes* [25,26,27]. For example, plant-derived phenolic compounds have been shown to suppress ROS production, modulate antioxidant enzymes, and inhibit inflammatory mediators in *C. acnes*-stimulated models [24]. Similarly, polyphenols and related bioactive molecules target multiple aspects of acne pathophysiology, including microbial growth, sebum regulation, and inflammatory signaling [21]. Recent research highlights the potential of novel antioxidant compounds and formulations in mitigating *C. acnes*-induced inflammation, overcoming antibiotic resistance, and restoring skin homeostasis [17,18,19,20]. Considerable attention is paid to phytochemicals as emerging therapeutics for acne vulgaris [17]. This study aims to further explore these relationships, focusing on the antibacterial and therapeutic potential of bearberry leaf extracts.

An increasing body of evidence supports the potential of plant extracts and plant-derived metabolites to be used as complementary or alternative approaches in acne management [20]. In this context, *Arctostaphylos uva-ursi* (bearberry) has recently attracted attention due to its reported antibacterial and antiproliferative properties [28,29,30], although data on its activity against skin-associated microorganisms implicated in acne pathogenesis are limited [31]. Bearberry-based herbal preparations are commonly formulated as infusions, tinctures, dry extracts, serums, creams, tonics, or masks, indicating their versatility for topical application. This warrants further investigations into the chemical profile and biological activity of bearberry leaves using extraction solvents with varying polarity. This study aimed to (i) analyze and compare the chemical composition of water and ethanolic extracts of *Uvae ursi folium* used as pharmacopoeial raw material; (ii) evaluate the cytotoxic, antioxidant, and antibacterial properties of the extracts; and (iii) determine their therapeutic index and potential suitability for subsequent in vivo investigations.

## 2. Results

### 2.1. Characteristics and Differentiation of the Chemical Composition of Bearberry Dry Extracts

The dry water and ethanol extracts had different chemical profiles (Table 1). The contents of arbutin, hydroquinone, picein, methylarbutin, corilagin, and hyperoside were statistically significantly higher in the water extract (WE), compared to the ethanolic extract (EE), while the total flavonoid content was similar in both extracts. In contrast, the content of penta-O-galloyl-β-d-glucose (PGG) and the total phenolic content were statistically significantly higher in the ethanolic extract than in the water extract. No oleanolic and ursolic acids were detected in the aqueous solutions, as they are practically insoluble in water, mainly due to their highly hydrophobic chemical structure [32,33].

**Figure 1:**
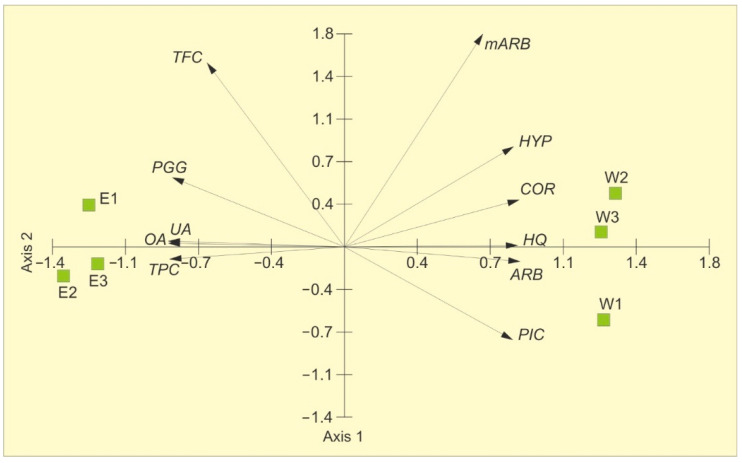
PCA ordination on the basis of the chemical composition of bearberry water extracts (W 1–3) and ethanolic extracts (E 1–3). ARB—arbutin, HQ—hydroquinone, PIC—picein, mARB—methylarbutin, COR—corilagin, PGG—penta-O-galloyl-β-d-glucose, HYP—hyperoside, OA—oleanolic acid, UA—ursolic acid, TPC—total phenolic content, TFC—total flavonoid content.

The variability in the chemical characteristics is presented in Figure 1. The principal component analysis (PCA) showed the distribution of samples along the first axis. The two PCA axes account for 97.44% of the total variance, with 90.27% explained by the first axis, indicating that two principal components sufficiently describe the samples (Table 2). The contents of arbutin, hydroquinone, picein, methylarbutin, and corilagin in the extracts positively correlate with the first axis, whereas PGG, hyperoside, oleanolic acid, ursolic acid, total phenolic content, and total flavonoid content are negatively correlated. The concentrations of methylarbutin, hyperoside, and total flavonoid content are positively correlated with the second axis. Two distinct groups are evident: water extracts on the right and ethanolic extracts on the left. The water extracts are characterized by the highest contents of arbutin, hydroquinone, picein, methylarbutin, and corilagin and the lowest concentrations of total phenolic compounds, total flavonoids, and PGG, compared to the ethanolic extracts.

### 2.2. Antioxidant Activity

The results of the analysis showed a variation in the antioxidant activity parameters in the extracts (Table 3). The FRAP parameter was statistically significantly higher (t = −9.33, *p* < 0.001) in the ethanolic extract (EE), compared to the water extracts (WE). A similar relationship was recorded in the case of DPPH (t = −8.98, *p* < 0.001). However, no statistically significant differences were found between the mean values of ABTS (t = −0.18, *p* = 0.854).

**Figure 2:**
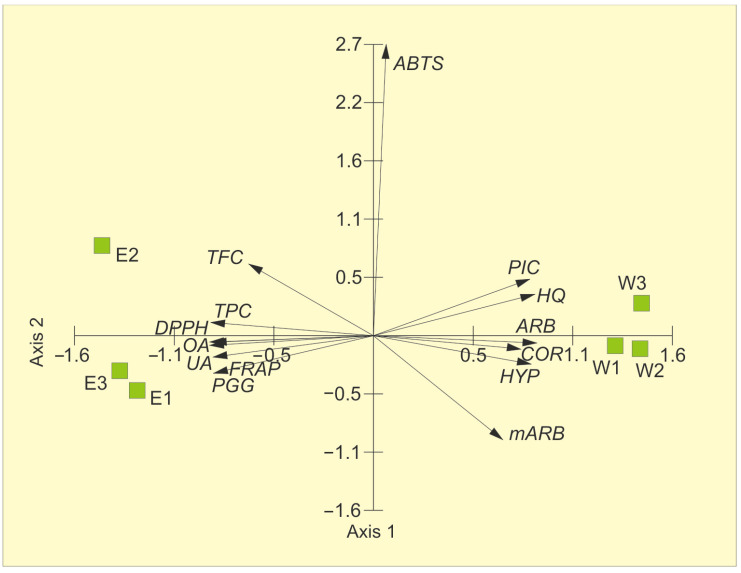
Results of PCA based on the chemical composition of bearberry water extracts (W 1–3) and ethanolic extracts (E 1–3) and antioxidant activity parameters. ARB—arbutin, HQ—hydroquinone, PIC—picein, mARB—methylarbutin, COR—corilagin, PGG—penta-O-galloyl-β-d-glucose, HYP—hyperoside, OA—oleanolic acid, UA—ursolic acid, TPC—total phenolic content, TFC—total flavonoid content, FRAP—ferric reducing antioxidant power, ABTS—ABTS^•+^ scavenging activity, DPPH—DPPH^•^ scavenging activity.

Figure 2 presents the principal component analysis of the chemical composition of the bearberry water and ethanolic extracts and their antioxidant activity parameters. The first two axes explained 93.6% of the variability (84.62%—Axis 1 and 8.97%—Axis 2). Arbutin, hydroquinone, picein, methylarbutin, corilagin, and hyperoside, i.e., metabolites characteristic of water extracts, were positively correlated with Axis 1, whereas negative correlations were found for TPC, UA, OA, and PGG. In turn, Axis 2 was positively correlated with ABTS and negatively correlated with mARB. The proximity of TPC, OA, and UA vectors on the left side of the ordination space to EE samples, which contain greater quantities of the aforementioned molecules than WE, proves their antioxidant activity (Figure 2, Table 4). The opposite direction of arbutin, hydroquinone, picein, methylarbutin, corilagin, and hyperoside vectors for WE indicates their significantly lesser role as antioxidants (Figure 2).

### 2.3. Cytotoxic Activity of Bearberry Extracts

**Figure 3:**
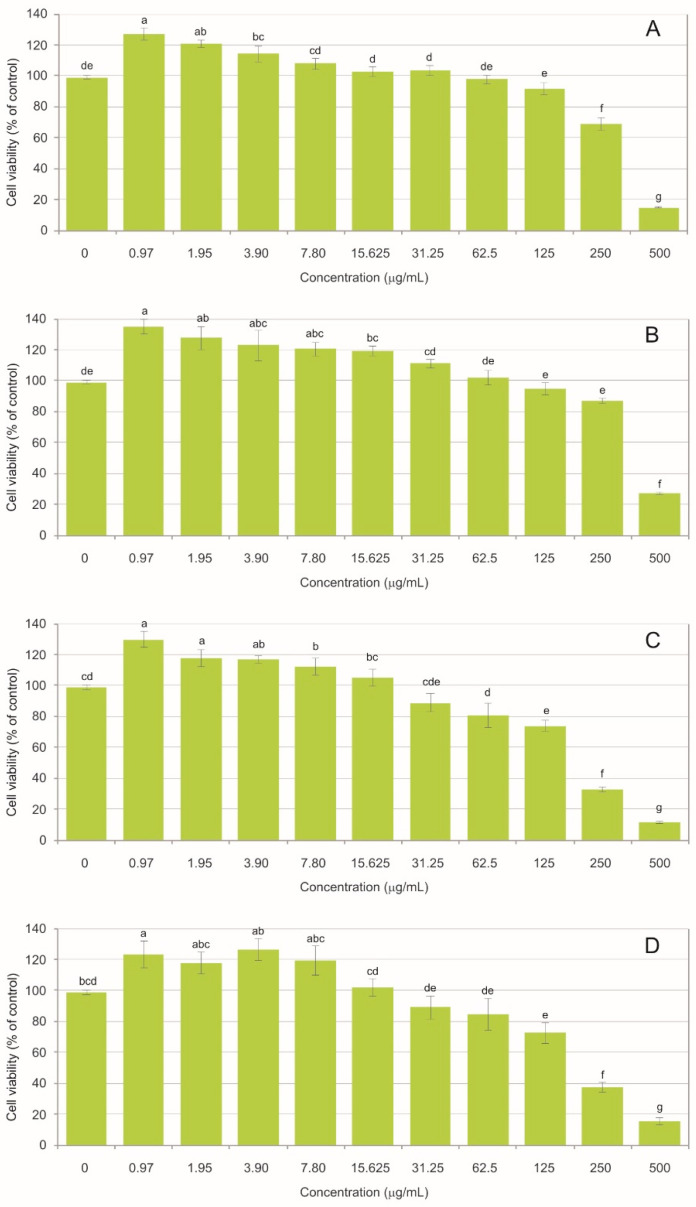
Cytotoxic activity of bearberry extracts towards normal human skin fibroblasts. (**A**)—water extract (24 h), (**B**)—ethanolic extract (24 h), (**C**)—water extract (48 h), (**D**)—ethanolic extract (48 h). The values designated by different small letters are significantly different, Tukey test, *p* < 0.05.

Both the water (WE) and ethanolic (EE) extracts of *Arctostaphylos uva-ursi* exhibited concentration- and time-dependent cytotoxicity toward BJ fibroblasts. At the highest concentrations, cell viability declined markedly (Figure 3). For the water extract, cell viability was reduced to 15.08% (±0.40) after 24 h and 11.77% (±0.72) after 48 h. A comparable trend was found for the ethanolic extract, where viability reached 20.28% (±4.93) after 24 h and declined to 15.63% (±2.26) after 48 h. These results indicate a progressive cytotoxic effect, regardless of the extraction solvent used. The non-toxic threshold, defined as cell viability not lower than 70% relative to the control, was observed at 125 µg/mL. For the water extract, viability was 92.04% (±3.90) after 24 h and decreased to 74.00% (±3.83) after 48 h. A similar pattern was recorded for the ethanolic extract, with viability of 94.93% (±4.17) after 24 h and 72.61% (±6.66) after 48 h, demonstrating that prolonged exposure shifts borderline concentrations toward cytotoxicity. A gradual increase in the cell number was observed with the decreasing extract concentration, indicating stimulation of proliferation. This trend was evident from 15.625 µg/mL for both incubation times and for both extract types. The strongest proliferative response was observed at the lowest concentration of 0.97 µg/mL. The ethanolic extract produced viability values of 135.08% (±6.55) after 24 h and 123.29% (±8.61) after 48 h, whereas the water extract resulted in cell viability of 127.10% (±4.18) after 24 h and 129.90% (±5.45) after 48 h.

**Figure 4:**
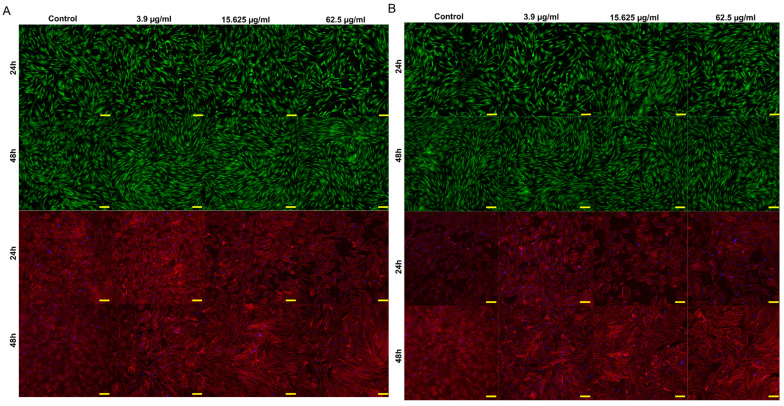
Confocal laser scanning microscopy (CLSM) images of BJ fibroblasts treated with extracts. (**A**) Cells treated with WE extract and (**B**) cells treated with EE extract. Live/dead staining (green—viable cells, red—dead cells) was performed after 24 and 48 h of incubation with the respective extract. Cell nuclei exhibited blue fluorescence (Hoechst 33342), while the cytoskeleton showed red fluorescence (Alexa Fluor 635) after 3 and 5 days of culture with the respective extract. Images were acquired at 100× magnification; scale bar = 100 µm.

The results demonstrate a biphasic hormetic response, where high concentrations induced cytotoxicity while low concentrations promoted fibroblast proliferation. The concentrations of 62.5, 15.625, and 3.9 µg/mL were used to represent three biologically relevant response levels identified in the viability assay (Figure 3). The highest concentration corresponded to a subcytotoxic range enabling visualization of early cellular damage. In turn, the intermediate concentration represented the transition zone between toxic and proliferative responses, and the lowest concentration reflected the proliferative range with preserved cell morphology. This approach allowed evaluation of morphological alterations across distinct biological responses to the extracts, as illustrated in the CLSM images (Figure 4A for WE and Figure 4B for EE).

Live/dead staining revealed predominantly viable cells, with green fluorescence clearly prevailing in all analyzed variants and no red-fluorescent dead cells. The cells maintained normal morphology and adhesion, exhibiting a typical fibroblast-like elongated shape and a uniform distribution across the culture surface. Fluorescent staining of nuclei and the cytoskeleton demonstrated normal cellular organization. Hoechst staining showed intact regularly shaped nuclei without signs of chromatin condensation or fragmentation, whereas cytoskeletal staining revealed well-developed filamentous structures and preserved cell spreading. No structural damage, cell shrinkage, or cytoskeletal collapse was detected, confirming the good cytocompatibility of the tested extract concentrations.

### 2.4. Antibacterial Activity of Bearberry Dry Extracts

The antibacterial activity of the extracts was evaluated using the agar diffusion method, and inhibition zone diameters were measured to assess microbial susceptibility. As shown in Table 5, EE exhibited stronger antibacterial activity than WE, with the highest susceptibility observed in Gram-positive *Staphylococcus* strains. Both extracts exhibited antibacterial effects, as all tested Gram-positive strains—both aerobic and microaerophilic—were inhibited, with inhibition zones ranging from 13.10 to 24.20 mm. More specifically, for *Staphylococcus* spp., the diameters of inhibition zones ranged from 17.03 to 24.20 mm, whereas smaller inhibition zones, ranging from 13.10 to 16.30 mm, were recorded for microaerophilic bacteria from the *Cutibacterium*/*Propionibacterium* group. Sparfloxacin, a widely used reference antibiotic with well-documented efficacy, exhibited the highest antibacterial activity.

The diffusion tests in solid medium and measurements of the bacterial growth inhibition zones showed varying responses of the bacterial strains to the bearberry water (WE) and ethanolic (EE) extracts (Table 5). Student’s *t*-test results showed a significant effect of the different extracts on the bacterial growth inhibition in the case of *Cutibacterium acnes* ATCC 11827 (t = −4.29, *p* = 0.013), *Propionibacterium acnes* PCM 2334 (t = −3.24, *p* = 0.032), and *Staphylococcus epidermidis* ATCC 12228 (t = −13.36, *p* < 0.001). The bearberry extracts had no effect on the growth zone in *Staphylococcus aureus* ATCC 25923 (t = −2.37, *p* = 0.077) and *Propionibacterium acnes* PCM (t = 0.23, *p* = 0.829).

**Figure 5:**
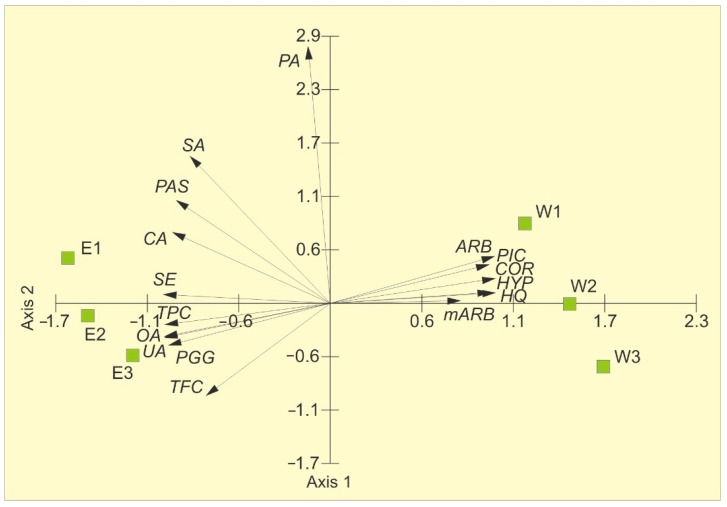
PCA ordination on the basis of the chemical composition of bearberry water extracts (W 1–3) and ethanolic extracts (E 1–3), and zones of bacterial growth inhibition induced by these extracts. CA—*C. acnes* ATCC 11827, PAS—*P. acnes* PCM 2334, PA—*P. acnes* PCM 2400, SA—*S. aureus* ATCC 25923, SE—*S. epidermidis* ATCC 12228.

The results of the principal component analysis of the chemical composition of the WE, EE, and bacterial growth inhibition zones are presented in Figure 5. The first two axes explained 93.3% of the variability (82.35%—Axis 1 and 10.95%—Axis 2). Arbutin, hydroquinone, picein, methylarbutin, corilagin, and hyperoside, i.e., metabolites characteristic of water extracts, were positively correlated with Axis 1, whereas negative correlations were found for TPC, UA, and OA. Axis 2 positively correlated with arbutin, picein, PAS, SA, and PA, and negatively correlated with PGG and TFC. The proximity of TPC, UA, and OA vectors along the first axis, which were more abundant in EE than in WE, and the growth inhibition zones of *C. acnes* ATCC 11827, *P. acnes* PCM, *S. aureus* ATCC 25923, and *S. epidermidis* ATCC 12228 proves the bactericidal effect of these extracts on the studied microorganisms (Figure 5, Table 6). The negative correlation between arbutin, hydroquinone, picein, methylarbutin, corilagin, and hyperoside found for WE and the growth inhibition zones of the bacterial strains indicates their significantly lesser role as antibacterial molecules (Figure 5).

### 2.5. Antimicrobial Effect of Bearberry Extracts Against Selected Strains

The antibacterial activity of WE and EE was evaluated using the Minimum Inhibitory Concentration (MIC), MBC/MIC ratios, and the Therapeutic Index (TI), which are key parameters in microbiology for assessing the efficacy and safety of antimicrobial agents (Table 7). MIC represents the lowest concentration of a compound that inhibits visible bacterial growth, while MBC is the lowest concentration that results in bacterial death. The MBC/MIC ratio provides insight into the mode of action: ratios close to 1–4 indicate bactericidal activity, whereas higher ratios suggest bacteriostatic effects. Therapeutic Index (TI) is the ratio of a cytotoxic concentration (IC50) to MIC. Higher TI values are desirable, as they reflect a wider safety margin, whereas low TI values indicate a higher risk and a narrow therapeutic window.

The data show that EE consistently demonstrated greater antibacterial potency than WE, as reflected by the lower MIC values (EE: 3.15–12.5 µg/mL; WE: 12.5–50 µg/mL), indicating stronger growth inhibition across all tested strains. The MBC/MIC ratios suggested that both extracts were primarily bacteriostatic, with EE displaying slightly higher bactericidal potential against *Propionibacterium acnes* PCM 2334 and PCM 2400. The low MIC values and strong bactericidal activity confirmed the high efficacy of sparfloxacin, used here as a reference antibiotic, and provided a reliable benchmark for evaluating the antibacterial potential of the tested extracts.

The high TI values further highlight the selectivity and potential safety of EE. They ranged from 21.57 to 43.13, and were consistently higher than those for WE (5.33–10.70). The highest TI was observed for *P. acnes* PCM 2334, indicating a favorable combination of strong antibacterial activity and a wide safety margin.

## 3. Discussion

The biological activity and pharmacological relevance of herbal preparations, such as dry extracts, strongly depend on the concentration and profile of phytochemicals. While the characteristics of the native plant material (e.g., genetic predisposition for secondary metabolite production, plant origin and growing conditions, and post-harvest processing) play a role, the concentration of bioactive compounds in dry extracts is significantly influenced by the extraction solvent [1,34]. Therefore, selecting an appropriate solvent capable of efficiently extracting physiologically relevant molecules ensures improved therapeutic potential of herbal formulations [35,36,37].

In this study, the total phenolic content was significantly higher in all ethanolic extracts than in the water extracts (Table 1), which indicates that ethanol enhances the extraction of a wider spectrum of minor unidentified phenolic constituents, especially moderately polar and less polar compounds that were not monitored in this study but contributed to the total phenolic content.

*Arctostaphylos uva-ursi* leaves contain high levels of phenolic compounds, particularly arbutin, the principal bioactive constituent responsible for the biological activity of bearberry preparations [5]. Arbutin may undergo hydrolysis to hydroquinone, a redox-active compound modulating cellular oxidative balance, which explains the dual cellular response observed in the present study. Both water and ethanolic extracts exhibited pronounced concentration- and time-dependent cytotoxicity toward BJ fibroblasts. At higher concentrations, cell viability decreased markedly, and the effect intensified after prolonged exposure. This behavior is consistent with the activity of hydroquinone, which induces oxidative stress, mitochondrial dysfunction, and loss of metabolic activity in fibroblasts [38]. The progressive decrease in viability between 24 h and 48 h observed in our experiments further supports cumulative oxidative damage as the primary mechanism of toxicity. In contrast, lower extract concentrations increased cell viability above control levels. Similar effects have been reported for arbutin, which promotes proliferation and migration of dermal fibroblasts at non-toxic concentrations [39]. Such a biphasic response is characteristic of redox-active phytochemicals and reflects hormesis, where moderate levels of reactive oxygen species act as signaling molecules activating adaptive cellular pathways, whereas excessive ROS induce cell death [40]. The shift from proliferative to cytotoxic response with increasing concentrations indicates that the biological effect of *A. uva-ursi* extracts is governed primarily by intracellular oxidative balance. At low concentrations, phenolic constituents stimulate cellular metabolism and growth, while at high concentrations hydroquinone-mediated oxidative stress results in cellular damage. This mechanism explains both the increased viability at low doses and the strong cytotoxicity observed at higher concentrations. These findings are consistent with previous reports on responses to arbutin-containing plant extracts and confirm a concentration-dependent redox-mediated mode of action in fibroblasts. The fluorescence imaging results further support the viability assay findings. Live/dead staining revealed exclusively viable cells and no red fluorescence, indicating preserved membrane integrity at the tested concentrations. Additionally, nuclear and cytoskeletal staining confirmed normal cellular organization, with no chromatin condensation, fragmentation, or cytoskeletal disruption. Within the selected concentration range, the *A. uva-ursi* extracts did not induce structural damage to fibroblasts, consistent with previous reports showing that non-toxic concentrations of arbutin promote fibroblast survival and cellular activity [39], whereas cytotoxic effects occur only at higher doses associated with hydroquinone-induced oxidative stress [38]. Therefore, the biological response of cells depends primarily on concentration-dependent redox activity rather than direct membrane toxicity, which is characteristic of ROS-mediated hormetic responses [40].

In recent years, increasing attention has been focused on the use of phytochemicals and plant extracts as potential adjunctive therapies in the management of acne vulgaris, primarily due to their documented antibacterial, anti-inflammatory, and antioxidant properties, along with the lower incidence of their adverse effects, compared to conventional antibiotics and synthetic drugs [41,42,43,44]. The present study demonstrated higher values of antioxidant parameters and greater antibacterial activity, indicated by the bacterial growth inhibition zone, MIC, and TI, when ethanol extracts were used (Table 5). These extracts were characterized by significantly lower arbutin and hydroquinone content, higher TPC and PGG levels, and the presence of UA and OA, which were not detected in the water extracts (Table 1).

Polyphenols are the main constituents in bearberry, and arbutin, a phenolic glucoside, may be considered the principal active marker, as it occurs in high concentrations in the leaves [45]. Hydroquinone may also be present or generated through arbutin degradation [5,46]. According to the 2018 monographs of the European Medicines Agency (EMA), arbutin-containing plant materials are used in the treatment of urinary tract infections. The Commission regulates the arbutin content at a minimum level of 7% in the raw material intended for therapeutic use. These metabolites exhibit antibacterial activity against urinary tract pathogens and anti-inflammatory effects [46,47,48]. Infusions of *Uvae ursi* leaves have been reported to be effective mainly against *E. coli*, *Pseudomonas aeruginosa*, *Proteus mirabilis*, and *Staphylococcus aureus* [8,49]. Moreover, water and ethanolic extracts of arbutin-containing plants are used as components of complex herbal medicinal products for urinary tract infections [49]. However, the results of the present study did not demonstrate a positive correlation between arbutin content and antibacterial activity.

Recently, the interest in the use of medicinal plants for the treatment of infectious diseases has increased significantly. Therefore, the impact of total phenolics and total flavonoids on antibacterial and antioxidant activity has become the subject of numerous studies [50]. The role of total phenolics and total flavonoids present in extracts of *Achillea millefolium*, *Bergenia ciliata*, and *Aloe vera* in antibacterial activity has been underlined. A strong positive correlation between TPC and TFC and the inhibition of *Staphylococcus aureus* growth was reported by [51]. In turn, the antibacterial activity of phenolic compounds against *Streptococcus pyogenes* was demonstrated by Macé et al. [52]. The most common research models used to evaluate the antibacterial effects of phenolic-rich extracts from various medicinal plants involve methicillin-resistant *Staphylococcus aureus* (MRSA) and *Escherichia coli* strains [50,51,53,54,55]. Belew et al. [56] reported the antibacterial activity of polyphenol- and flavonoid-rich *Rhus vulgaris* extracts against *Salmonella typhimurium* and *Klebsiella pneumoniae*. Moreover, several authors have highlighted the role of polyphenols, particularly galloyl derivatives and flavonoids, as key contributors to antibacterial and antibiofilm effects [42,57]. These findings support our observation that extracts richer in total phenolics induced larger growth inhibition zones and exhibited more favorable therapeutic indices.

The present results demonstrated the absence of UA and OA in the water extracts, which may explain the observed variability in the chemical composition and the antioxidant and antibacterial activities (Figure 2 and Figure 3). Previous studies have attributed a broad spectrum of biological activities to these compounds, including antidiabetic and anti-inflammatory properties [58,59], antioxidant effects [59,60], as well as antifungal [61] and antimicrobial activities [62,63,64,65,66]. Verstraeten et al. [63] demonstrated the potential role of UA and OA in disrupting lipid membranes of methicillin-resistant *Staphylococcus aureus*, thereby enhancing antibiotic activity. Sekandi et al. [64] highlighted the activity of UA against *S. aureus*, *E. coli*, *Candida albicans*, and *Aspergillus flavus*. Li et al. [65] demonstrated the antibacterial potential of UA and investigated its underlying mechanisms in *Prunella vulgaris* L. against methicillin-resistant *Staphylococcus aureus*. In turn, Kurek et al. [66] showed that the pentacyclic triterpenoids UA and OA can modulate resistance to β-lactam antibiotics (ampicillin and oxacillin) in *Pseudomonas aeruginosa*, *S. aureus*, *S. epidermidis*, and *Listeria monocytogenes*. Moreover, these compounds exhibit anticancer potential, including the ability to induce programmed cell death, and have been proposed as promising agents in cancer prevention and treatment strategies [67,68]. Consistent with these reports, our findings indicate that both metabolites may substantially contribute to antibacterial activity. Accordingly, future investigations, particularly those focusing on antibacterial and anti-acne effects, should evaluate these compounds applied both individually and in combination.

Our results showed higher PGG content in the ethanolic extracts (Table 1) and a correlation between the PGG levels and the inhibition zones for CA and SE (Figure 4). This suggests a potential role of these compounds in antioxidant and antibacterial activities (Figure 2 and Figure 3). PGG has been reported to have multiple biological activities, indicating its great potential for use in the therapy and prevention of major diseases, including cancer and diabetes [69,70,71]. In turn, studies on *Fomitella fraxinea* indicate that PGG exerts anti-photoaging effects both in vitro and in vivo through the suppression of PAK1 and JNK1 kinase activities and may therefore be useful in the prevention of skin aging [72]. Furthermore, the antibacterial activities of four galloylglucoses isolated from *Paeonia officinalis* leaves against multidrug-resistant strains of *E. coli* and *K. pneumoniae* were reported by Masota et al. [73].

The present findings demonstrate that the individual components of bearberry extracts are highly active, but solvents determine their different chemical compositions and quantitative relationships between components. It should be assumed that their activity cannot be attributed to any single molecule, but rather indicates interactions within the extract. Medicinal plant extracts frequently outperform isolated constituents at equivalent doses, and complex extracts show stronger antibacterial effects than those expected from individual components alone [74,75,76]. As reported by Galma et al. [75], crude extracts of *Cucumis prophetarum* showed slightly higher antibacterial activity against multiple bacteria than isolated compounds. The enhanced activity was attributed to synergistic interactions among phytochemicals present in the extracts, displaying slightly higher antibacterial activity compared with isolated compounds. In turn, Donkor et al. [77] observed considerably lower MICs against all tested microorganisms in combined extracts than in individual extracts. It is generally agreed that combinations of multiple antimicrobial agents can result in varying effects depending on their composition and concentration [78].

Despite the promising anti-acne potential associated with the antimicrobial activity of bearberry extract phytochemicals, further toxicological studies are required to evaluate the potential risks associated with the side effects of bioactive constituents, especially hydroquinone. Synthetic hydroquinone, commonly used for the treatment of hyperpigmentation, has been linked in some studies to several adverse effects, such as damage to lipid cell membranes, inhibition of nucleic acid synthesis, skin irritation, induction of inflammation, and an increased risk of post-inflammatory hyperpigmentation [48,79,80]. However, the presence of co-occurring compounds in complex mixtures of natural origin may significantly alter the behavior of hydroquinone and potentially limit its negative effects; thus, more detailed studies are warranted in this area.

Previous investigations have also shown that *A. uva-ursi* extracts possess anti-biofilm activity against *C. acnes*, which is highly relevant in acne pathogenesis [31]. Biofilm formation represents a critical virulence factor, enhancing bacterial resistance to antimicrobial treatment and contributing to the chronic and recurrent nature of acne lesions [81]. The ability of bearberry leaf extracts to inhibit biofilm formation and promote biofilm disruption suggests that their mechanism of action extends beyond simple growth inhibition, targeting structured bacterial communities that are more resistant to conventional therapies [31,81]. Although not addressed in this paper, these findings will be reviewed in a separate study.

The relatively strong inhibitory effect against *S. epidermidis* ATCC 12228 should be interpreted with caution, as this strain is a commensal member of the skin microbiota contributing to skin homeostasis. Therefore, its excessive suppression may be undesirable given the potential disruption of microbial balance. At the same time, the present results were obtained under in vitro conditions, which do not fully reflect the complexity of the skin ecosystem, including microbial interactions and host factors. Consequently, the observed activity may not directly translate into in vivo effects. Future studies should assess the selectivity of the tested compounds and their impact on the skin microbiome to ensure a more targeted and microbiome-friendly therapeutic approach.

Comparable antimicrobial effects have been described for other plant extracts rich in phenolic compounds and flavonoids, key groups of secondary metabolites known to inhibit bacterial growth, disrupt cell membranes, interfere with enzymatic systems, and reduce pathogen adhesion [42,43,46,82]. Several studies have demonstrated that polyphenols and flavonoids can effectively suppress the proliferation of *C. acnes* and other skin-associated bacteria, such as *S. epidermidis* and *S. aureus*, often with lower cytotoxicity toward host cells than conventional synthetic agents [42,43,44,82].

Moreover, literature reviews emphasize that plant-derived antibacterial extracts frequently exhibit multitarget mechanisms of action, combining direct antimicrobial effects, inhibition of biofilm formation, and modulation of skin inflammatory responses [41,42,43,44,82]. Such pleiotropic activity makes them promising candidates for incorporation into dermatological and cosmetic formulations intended for acne management. Their antioxidant properties may further support tissue repair and reduce oxidative stress within acne lesions, thereby contributing to improved therapeutic outcomes [83].

Taking into account the antibacterial activity of polyphenols, UA, OA, and PGG documented in the literature, these groups of metabolites determine the antibacterial properties against the analyzed strains.

## 4. Materials and Methods

### 4.1. Chemicals

The extraction solvent, i.e., ethyl alcohol (96.6%), was obtained from Avantor Performance Materials (Gliwice, Poland). Chromatographic standards, i.e., β-arbutin (≥98%), corilagin (≥98%), hydroquinone (≥99%), hyperoside (≥97.0%), methylarbutin (≥97%), oleanolic acid (≥97%), pentagalloylglucose (≥96%), and picein (≥98%), were purchased from Sigma-Aldrich (St. Louis, MO, USA), whereas ursolic acid (≥98.5%) was obtained from Supelco (Bellefonte, PA, USA). Gradient grade HPLC elution components, i.e., formic acid (≥98%) and acetonitrile (≥99.9%), were purchased from Supelco (Bellefonte, PA, USA) and Sigma-Aldrich (St. Louis, MO, USA), respectively. Reagents for spectrophotometric determinations of total phenolic content and antioxidant activity, i.e., Folin & Ciocalteu’s phenol reagent (p.a.), aluminum chloride (AlCl_3_) (99%), 2,2-diphenyl-1-picrylhydrazyl (DPPH) (p.a.), and 2,2′-azino-bis(3-ethylbenzothiazoline-6-sulfonic acid) diammonium salt (ABTS) (≥98%), were obtained from Sigma-Aldrich (St. Louis, MO, USA). 2,4,6-Tris(2-pyridyl)-s-triazine (TPTZ) (≥99.0%) was purchased from Supelco (Bellefonte, PA, USA). Standards for spectrophotometric determinations, i.e., gallic acid (≥98%), quercetin (≥95%), and (±)-6-hydroxy-2,5,7,8-tetramethylchromane-2-carboxylic acid (Trolox) (97%), were obtained from Sigma-Aldrich (St. Louis, MO, USA). All other reagents were of analytical or higher grade.

### 4.2. Plant Material

The field study was carried out in dense bearberry patches located in a pine forest (N51 45.963; E22 13.202) in the Wysoczyzna Żelechowska Region (Eastern Poland) in August 2020. Three samples of plant material (40 g leaf fresh weight each) were collected for the phytochemical analyses. After collection, the plant material was placed in a refrigerator and transported to the laboratory, where the leaves were dried at room temperature in the laboratory. The *A. uva-ursi* specimens used in the study were identified by Anna Rysiak, a taxonomist from Maria Curie-Skłodowska University in Lublin. The reference material (4404P) was deposited in the collection of the Botanic Garden of Maria Curie-Skłodowska University in Lublin.

### 4.3. Preparation of Dry Extracts

Prior to extraction, *A. uva-ursi* leaves were powdered in a laboratory knife mill to obtain a homogeneous powder passing through a 0.5 mm sieve. The plant powder (5 g) was weighed on a laboratory balance and extracted sequentially three times using successive portions of water or ethanol (96.6%, *v*/*v*): 200 mL (60 min), 50 mL (30 min), and 50 mL (30 min). The extraction was conducted in a thermostatically controlled ultrasonic water bath set at 45 °C. Between each extraction stage and after the final step, centrifugation was applied to separate the supernatants from the insoluble residues. The combined supernatants from each extraction stage were passed through Whatman (grade 1) filter paper.

To obtain the dry water extract (WE), the filtrate was frozen at −50 °C and lyophilized. The dry ethanolic extract (EE) was prepared by removing the solvent under reduced pressure using a rotary evaporator. After drying, the extracts were pulverized using a mortar and pestle and stored in the dark at −20 °C in sealed foil bags until analysis.

To determine their phytochemical profile, the dry extracts were re-dissolved in water (WE) or ethanol (EE), followed by vortexing (30 s) and incubation for 5 min at 45 °C in an ultrasonic water bath.

### 4.4. Determination of the Phytochemical Profile of Dry Extracts

#### High-Performance Liquid Chromatography

The qualitative and quantitative profiling of constituents present in barberry dry extracts was carried out using a Varian ProStar high-performance liquid chromatography (HPLC) system equipped with a UV–VIS detector (Varian Inc., Walnut Creek, CA, USA).

Before chromatographic analysis, re-dissolved extracts were passed through 0.22 μm syringe membrane filters to remove potential impurities. A sample volume of 20 μL was loaded into the chromatographic system. Compound separation was performed on a reversed-phase Gemini C18 column (5 μm particle size, 110 Å pore size, 250 mm × 4.6 mm; Phenomenex, Torrance, CA, USA) thermostatted at 25 °C. The elution system consisted of solvent A (0.1% (*v*/*v*) formic acid in water) and solvent B (0.1% (*v*/*v*) formic acid in acetonitrile). The mobile phase was delivered at a constant flow rate of 1.0 mL/min. For the determination of arbutin, hydroquinone, hyperoside, corilagin, pentagalloylglucose, methylarbutin, and picein, the following gradient program was applied: 0–5 min, 96% A; 30 min, 78% A; 45 min, 75% A; 50–55 min, 0% A; and 60–65 min, 96% A to allow column re-equilibration. A separate gradient elution protocol was implemented for the analysis of oleanolic acid and ursolic acid: 0 min, 5% B; 20 min, 80% B; 35 min, 85% B; 40–45 min, 100% B; 50 min, 5% B; and 65 min, 5% B (column re-equilibration).

Arbutin, hydroquinone, picein, methylarbutin, corilagin, and pentagalloylglucose were monitored at 280 nm, hyperoside at 350 nm, and oleanolic acid and ursolic acid at 210 nm. Quantitative evaluation was conducted using external standard calibration curves. Validation parameters of the HPLC method were provided as Supplementary Materials (Table S1).

### 4.5. Spectrophotometric Assessments

#### 4.5.1. Total Phenolic Content

The overall phenolic content of the dry extracts was evaluated spectrophotometrically using the Folin–Ciocalteu colorimetric assay, based on the procedure described by Singleton and Rossi [83], with modifications enabling analysis in a 96-well microplate system.

Prior to analysis, each dry extract was diluted at a ratio of 1:4 using the corresponding extraction solvent. A 10 μL aliquot of the diluted solution was transferred to a microplate well and combined with 100 μL of distilled water, followed by the addition of 20 μL of Folin–Ciocalteu reagent, previously diluted fivefold with distilled water. After an initial reaction period of 3 min, 100 μL of a 10% (*w*/*v*) Na_2_CO_3_ solution was added to initiate chromophore development. The reaction mixtures were subjected to orbital shaking (100 rpm) for 30 s and subsequently incubated at ambient temperature for 30 min.

The absorbance was recorded at 765 nm using a microplate reader, with appropriate reagent blanks applied for background correction. Quantitative determination was performed by reference to an external calibration curve constructed with gallic acid as a standard. The results were expressed as milligrams of gallic acid equivalents (GAE) per gram of dry extract.

#### 4.5.2. Total Flavonoid Content

The total flavonoid content in the extracts was quantified using a colorimetric method based on formation of a complex with aluminum ions (Al^3+^) [Lamaison], with modifications enabling analysis in a 96-well microplate system.

For the assay, 150 µL of each extract, previously diluted 1:4 (*v*/*v*) with the corresponding extraction solvent (water or ethanol for WE and EE, respectively), was combined with an equal volume (150 µL) of a 3% AlCl_3_ solution (*w*/*v*) prepared in water for WE or ethanol for EE. The mixtures were incubated at room temperature for 30 min to allow complete formation of the flavonoid–Al^3+^ complexes. Following incubation, the absorbance was recorded at 430 nm using a microplate reader, with appropriate blanks for correction. The flavonoid content was calculated from an external calibration curve constructed with quercetin and expressed as quercetin equivalents (QE) per gram of dry extract.

#### 4.5.3. Ferric Reducing Antioxidant Power

Ferric reducing antioxidant power (FRAP) of dry extracts was evaluated using a colorimetric assay based on the method described by Benzie and Strain [84], with modifications enabling analysis in a 96-well microplate system. The working reagent was freshly prepared by combining 0.3 M sodium acetate buffer (pH 3.6), 10 mM 2,4,6-tripyridyl-s-triazine (TPTZ), and 20 mM ferric chloride in a volumetric ratio of 10:1:1 (*v*/*v*/*v*).

Prior to analysis, the re-dissolved extracts were diluted fourfold with the corresponding extraction solvent (water or ethanol for WE and EE, respectively). A 4 µL aliquot of the diluted extract was mixed with 40 µL of the prepared reagent and 200 µL of distilled water in a microplate well. The reaction mixtures were incubated at ambient temperature for 30 min to allow complete reduction of the ferric–TPTZ complex.

Absorbance was recorded at 593 nm using a microplate reader, with appropriate blanks for correction. The reducing capacity was quantified relative to a Trolox standard curve and expressed as milligrams of Trolox equivalents per gram of dry extract.

#### 4.5.4. ABTS and DPPH Scavenging Activities

Scavenging activities against the ABTS radical cation and the DPPH radical were determined based on the decolorization assays according to the methods developed by Re et al. [85] and Brand-Williams et al. [86], respectively. Modifications enabling analysis in a 96-well microplate system were applied. Before analysis, the re-dissolved extracts were diluted twenty-fold with the corresponding extraction solvent (water or ethanol for WE and EE, respectively).

Reaction reagents were prepared strictly according to the original procedures [86,87]. The samples (5 µL) were mixed with 300 µL of ABTS or DPPH reagent and left to stand for 120 min at room temperature. Absorbance at 734 nm for the ABTS assay and 515 nm for the DPPH assay was recorded using a microplate reader, with appropriate blanks for correction. ABTS and DPPH scavenging activities were quantified relative to a Trolox standard curve and expressed as milligrams of Trolox equivalents per gram of dry extract.

### 4.6. Cytotoxic Activity

#### 4.6.1. Cell Culture Experiments

Normal human skin cells (BJ line, CRL-2522™, ATCC, Manassas, VA, USA) were cultured in Minimum Essential Medium containing Earle’s salts (EMEM, ATCC), supplemented with 10% fetal bovine serum (PAN Biotech, Aidenbach, Germany) and a 1% (*v*/*v*) antibiotic solution (100 U·mL^−1^ penicillin and 1000 μg·mL^−1^ streptomycin, Sigma-Aldrich Chemicals, Warsaw, Poland. The cells were maintained at 37 °C in a humidified atmosphere containing 5% CO_2_ and 95% air (Heraeus Cytoperm 2, Thermo Scientific, Waltham, MA, USA).

#### 4.6.2. Cell Viability

Cell viability was assessed according to the procedure described in detail in our previous study [87]. Extracts were initially dissolved in DMSO to obtain a stock solution (100 mg/mL). Serial dilutions were subsequently prepared in EMEM supplemented with 2% FBS to achieve final concentrations ranging from 0.97 to 500 µg/mL. Human fibroblasts (BJ) were seeded into 96-well plates at a density of 1 × 10^4^ cells per well in 100 µL of culture medium and incubated for 24 h at 37 °C in a humidified atmosphere containing 5% CO_2_. After incubation, the medium was replaced with 100 µL of the tested compounds at the indicated concentrations. Corresponding DMSO controls were included to exclude solvent-related cytotoxic effects. Following 24 h exposure, cell viability was evaluated using the MTT assay (Sigma-Aldrich, St. Louis, MO, USA). The tested solutions and DMSO controls were removed, and 100 µL of culture medium containing 1 mg/mL MTT was added to each well. After 3 h incubation, 100 µL of an SDS solution prepared in 0.01 M HCl (Avantor Performance Materials Poland S.A., Gliwice, Poland) was added, and the plates were incubated for an additional 12 h. Absorbance was measured at 570 nm using a microplate reader (BioTek Synergy, Winooski, VA, USA). Cell viability was expressed as a percentage of the negative control (cells cultured in medium without extracts), which was considered 100% viability.

#### 4.6.3. Fluorescence Staining and CLSM Imaging

For qualitative cytocompatibility assessment, fluorescence staining was performed as previously described [88], with minor modifications. For the Live/Dead assay, BJ fibroblasts were seeded under the same conditions as described for the MTT assay and, after attachment, exposed to the tested extracts for 24 h or 48 h. The cells were then stained using a Live/Dead double staining kit (Sigma-Aldrich Chemicals, Warsaw, Poland) according to the manufacturer’s protocol and observed using a confocal laser scanning microscope (CLSM, Olympus FluoView equipped with FV1000, Shinjuku, Japan).

For visualization of the cytoskeleton and nuclei, BJ fibroblasts were seeded at a density of 3 × 10^4^ cells/mL. After 24 h, the culture medium was replaced with the tested extracts and the cells were cultured for 3 and 5 days. After 3 days of incubation, the extracts were replaced with fresh solutions and the incubation was continued until day 5. Following incubation, the cells were fixed with 3.7% formaldehyde for 10 min at room temperature and washed twice with PBS. The samples were then blocked with 1% bovine serum albumin (BSA) for 30 min, permeabilized with 0.2% Triton X-100 for 5 min, and rinsed twice with PBS. F-actin was stained with Alexa Fluor™ (Invitrogen Warsaw, Poland) 635-conjugated phalloidin, and cell nuclei were stained with Hoechst 33342. The samples were observed using a confocal laser scanning microscope (CLSM, Olympus FluoView equipped with FV1000, Shinjuku, Japan).

### 4.7. Antibacterial Activity

#### 4.7.1. Bacterial Strains and Culture Conditions

The antibacterial activity of WE and EE was evaluated against acne strains *Cutibacterium acnes* (formerly *Propionibacterium acnes*). The original nomenclature provided by culture collections (e.g., the Polish Collection of Microorganisms, PCM) has been retained in strain identifiers: *Cutibacterium acnes* ATCC 11827, *Propionibacterium acnes* PCM 2334, *Propionibacterium acnes* PCM 2400, *Staphylococcus aureus* ATCC 25923, and *Staphylococcus epidermidis* ATCC 12228. Aerobic strains (*Staphylococcus* spp.) were cultured on Mueller-Hinton (M-H) agar and in M-H broth, whereas microaerophilic strains (*Cutibacterium* and *Propionibacterium*) were cultured on Brain Heart Infusion (BHI) agar and in BHI broth. All cultures were incubated at 36 °C for 24 h under appropriate atmospheric conditions. Sparfloxacin, used as a positive control, was purchased from Pol-Aura Sp. z o.o., Morąg, Poland and selected as a clinically relevant antibiotic for skin pathogens.

#### 4.7.2. Preparation of Extracts

Dry water and dry ethanolic extracts were prepared, dissolved in DMSO, and then diluted to appropriate concentrations for antimicrobial testing. Standard controls were included in all microbial assays: negative controls (sterile broth), positive controls (bacterial growth), solvent (DMSO) controls, and, for liquid assays, additional extract color controls to prevent interference from the sample coloration.

#### 4.7.3. Agar Diffusion Assay (Zones of Inhibition)

The antibacterial activity of the extracts was initially assessed using the agar diffusion method. One hundred micrograms (stock 10 mg/mL) of each extract was applied onto agar plates inoculated with the test strains. The plates were incubated under strain-specific conditions for 24 h, and the diameters of inhibition zones were measured in millimeters. The experiments were performed in triplicate.

#### 4.7.4. Determination of Minimum Inhibitory Concentration (MIC)

MICs were determined using the broth microdilution method, with extract concentrations ranging from 800 to 3.15 µg/mL. McFarland standard 1 × 10^8^ CFU/mL of the respective strain was prepared. MIC was defined as the lowest concentration of extract that inhibited visible bacterial growth after 24 h incubation at 36 °C.

#### 4.7.5. Determination of Minimum Bactericidal Concentration (MBC) and MBC/MIC Ratio

Following MIC determination, aliquots from wells showing no visible growth were plated onto agar and incubated under optimal conditions for each strain. MBC was defined as the lowest extract concentration resulting in ≥99.9% reduction in viable bacteria. The MBC/MIC ratio was calculated to distinguish bactericidal (ratio ≤ 4) from bacteriostatic (ratio > 4) activity.

### 4.8. Statistical Analysis

Prior to the analyses, the assumptions of normality and homogeneity of variance were verified using the Shapiro–Wilk and Levene tests, respectively. One-way analysis of variance (ANOVA) and subsequent Tukey tests were used to compare the cytotoxic activity of bearberry extracts toward normal human skin fibroblasts, as well as growth inhibition zones (mm) resulting from the effects of sparfloxacin and bearberry water and ethanolic leaf extracts. In turn, Student’s *t*-test was used to compare the content of secondary metabolites in water and ethanolic bearberry leaf extracts. Differences were considered significant at *p* < 0.05. The statistical analyses were carried out using Statistica 6.0 software (Stat. Soft, Inc., Kraków, Polska). Principal component analysis (PCA) was performed to explain the relationships between the components of the examined bearberry extracts and antioxidant parameters and bacterial growth inhibition zones. Prior to the PCA, the data were centered and log-transformed. The analyses were carried out using the statistical package (MVSP) program version 3.1 [89].

## 5. Conclusions

The present study demonstrates higher values of antioxidant parameters and greater antibacterial activity of the bearberry dry ethanolic extract, measured by bacterial growth inhibition, MIC, and TI when it was used, compared to the dry water extract. The ethanolic extract exhibited significantly lower arbutin and hydroquinone content but higher total phenolic content and penta-O-galloyl-β-d-glucose levels, as well as the presence of ursolic acid and oleanolic acid, which were not detected in the water extract. The ethanolic extract exhibited higher antioxidant activity and the most favorable overall biological properties. The therapeutic index (TI) values were as follows: *Cutibacterium acnes* ATCC 11827 (10.70; 21.57), *Propionibacterium acnes* PCM 2334 (10.70; 43.13), *P. acnes* PCM (5.33; 21.57), *Staphylococcus aureus* ATCC 25923 (10.70; 21.57), and *S. epidermidis* ATCC 12228 (5.33; 10.78) for the water and ethanolic extracts, respectively. The high TI values indicated that the bearberry extract is a promising antibacterial agent and may be considered for in vivo testing. *Uva ursi folium* can be regarded as an interesting and valuable source for the pharmaceutical and cosmetic industries, especially given the rising antibiotic resistance in skin pathogens observed in recent years. Nevertheless, further investigations are necessary to elucidate the mechanism of action of bearberry dry extracts against pathogenic skin strains.

## Figures and Tables

**Table 1:** Content of secondary metabolites in water and ethanolic bearberry leaf extracts. WE—water extract, EE—ethanolic extract. Student’s *t*-test; values designated by different letters are significantly different (*p* < 0.05).

	WE		EE	
Metabolites	Mean	SD	Mean	SD
Arbutin (mg g^–1^)	191.00 ^a^	3.032	142.32 ^b^	2.291
Hydroquinone (mg g^–1^)	35.10 ^a^	1.480	19.81 ^b^	1.285
Picein (mg g^–1^)	4.06 ^a^	0.176	3.12 ^b^	0.141
Methylarbutin (mg g^–1^)	5.07 ^a^	0.296	4.54 ^b^	0.240
Corilagin (mg g^–1^)	3.32 ^a^	0.307	1.24 ^b^	0.108
PGG (mg g^–1^)	6.33 ^b^	0.401	10.11 ^a^	0.456
Hyperoside (mg g^–1^)	19.63 ^a^	0.956	16.00 ^b^	0.420
Oleanolic acid (mg g^–1^)	0.00 ^b^	0.000	2.33 ^a^	0.032
Ursolic acid (mg g^–1^)	0.00 ^b^	0.000	19.91 ^a^	0.225
Total phenolic content (mg GAE/g)	387.20 ^b^	8.70	439.43 ^a^	9.500
Total flavonoid content (mg QE/g)	6.34 ^a^	0.089	6.46 ^a^	0.073

**Table 2:** Results of PCA based on the secondary metabolite composition of water and ethanolic bearberry leaf extracts. (A) Eigenvalues and variance (%) explained by the first two PCA axes; (B) Loading components for each variable associated with the two axes.

	Axis 1	Axis 2
(A)	9.93	0.79
(B)	90.27	7.17
Arbutin	0.316	−0.045
Hydroquinone	0.312	0.005
Picein	0.303	−0.287
Methylarbutin	0.249	0.656
Corilagin	0.315	0.145
PGG	−0.31	0.214
Hyperoside	0.305	0.309
Oleanolic acid	−0.317	0.011
Ursolic acid	−0.317	0.018
Total phenolic content	−0.315	−0.038
Total flavonoid content	−0.247	0.567

**Table 3:** Antioxidant activity parameters of water (WE) and ethanolic (EE) extracts from bearberry leaves. FRAP—ferric reducing antioxidant power, ABTS—ABTS^•+^ scavenging activity, DPPH—DPPH^•^ scavenging activity; Student’s *t*-test; values designated by different letters are significantly different (*p* < 0.05).

	WE		EE	
	Mean	SD	Mean	SD
FRAP (mg TE g^–1^)	490.34 ^b^	8.68	538.92 ^a^	11.18
ABTS (mg TE g^–1^)	1415.09 ^a^	25.17	1411.90 ^a^	36.50
DPPH (mg TE g^–1^)	947.32 ^b^	20.14	1069.99 ^a^	31.01

**Table 4:** Results of PCA based on the chemical composition of bearberry extracts and antioxidant activity parameters. (A) Eigenvalues and variance (%) explained by the first two PCA axes; (B) Loading components for each variable associated with the two axes.

	Axis 1	Axis 2
(A)	11.84	1.26
(B)	84.62	8.97
Arbutin	0.29	−0.022
Hydroquinone	0.286	0.125
Picein	0.277	0.172
Methylarbutin	0.229	−0.316
Corilagin	0.288	−0.04
PGG	−0.283	−0.114
Hyperoside	0.28	−0.086
Oleanolic acid	−0.29	−0.019
Ursolic acid	−0.29	−0.03
Total phenolic content	−0.288	0.04
Total flavonoid content	−0.221	0.217
FRAP	−0.284	−0.065
ABTS	0.022	0.882
DPPH	−0.286	−0.02

**Table 5:** Growth inhibition zones (mm) resulting from the impact of bearberry water and ethanolic leaf extracts. Tukey test; values designated by different letters are significantly different (*p* < 0.05).

	Sparfloxacin		WE		EE	
	Mean	SD	Mean	SD	Mean	SD
*Cutibacterium acnes* ATCC 11827	24.00 ^c^	0.94	13.10 ^a^	0.79	15.60 ^b^	0.62
*Propionibacterium acnes* PCM 2334	24.03 ^c^	0.47	14.10 ^a^	0.75	16.30 ^b^	0.90
*Propionibacterium acnes* PCM 2400	26.33 ^b^	0.58	14.07 ^a^	0.60	13.97 ^a^	0.45
*Staphylococcus aureus* ATCC 25923	29.00 ^b^	1.15	19.03 ^a^	1.60	22.03 ^a^	1.50
*Staphylococcus epidermidis* ATCC 12228	32.10 ^c^	1.60	17.03 ^a^	0.76	24.20 ^b^	0.53

**Table 6:** Results of PCA based on the chemical composition of the bearberry extracts and zones of bacterial growth inhibition induced by the extracts. (A) Eigenvalues and variance (%) explained by the first two PCA axes. (B) Loading components for each variable associated with the two axes.

	Axis 1	Axis 2
(A)	13.18	1.75
(B)	82.35	10.95
Arbutin	0.271	0.123
Hydroquinone	0.272	0.026
Picein	0.263	0.116
Methylarbutin	0.213	−0.035
Corilagin	0.271	0.054
PGG	−0.268	−0.121
Hyperoside	0.263	0.009
Oleanolic acid	−0.273	−0.089
Ursolic acid	−0.274	−0.086
Total phenolic content	−0.273	−0.047
Total flavonoid content	−0.206	−0.273
*Cutibacterium acnes* ATCC 11827	−0.258	0.200
*Propionibacterium acnes* PCM 2334	−0.25	0.298
*Propionibacterium acnes* PCM 2400	−0.002	0.749
*Staphylococcus aureus* ATCC 25923	−0.229	0.410
*Staphylococcus epidermidis* ATCC 12228	−0.275	0.030

**Table 7:** MIC: minimal inhibitory concentration; MBC/MIC ratio: minimal bactericidal concentration/MIC ratio; TI: therapeutic index of WE and EE against selected strains.

	MIC [µg/mL]			MBC/MIC			TI	
	Sparfloxacin	WE	EE	Sparfloxacin	WE	EE	WE	EE
*Cutibacterium acnes* ATCC 11827	3.90	25	12.5	2	4	4	10.70	21.57
*Propionibacterium acnes* PCM 2334	1.95	25	6.25	2	2	2	10.70	43.13
*Propionibacterium acnes* PCM 2400	0.97	50	12.5	2	2	2	5.33	21.57
*Staphylococcus aureus* ATCC 25923	0.97	50	6.25	1	≥4	≥4	10.70	21.57
*Staphylococcus epidermidis* ATCC 12228	0.48	12.5	3.15	1	≥4	≥4	5.33	10.78

TI = IC_50_(48 h)/MIC.

## Data Availability

The data presented in this study are available on request from the corresponding authors.

## Supplementary Materials

The following supporting information can be downloaded at: https://www.mdpi.com/article/10.3390/molecules31081267/s1, Table S1: Basic validation parameters of HPLC method for bearberry extract phytochemicals analysis.
