# Supplementary material for: Chemical Characteristics and Antimicrobial Activity of Arctostaphylos uva-ursi (L.) Spreng. Extracts Against Skin-Associated Bacteria
Source: Molecules. 2026 Apr 12;31(8):1267. doi: 10.3390/molecules31081267 (PMC13119283; doi:10.3390/molecules31081267)
Supplement: Supplementary file 1 [file molecules-31-01267-s001.zip › molecules-4205788-supplementary.pdf]

Supplementary material

Table S1. Basic validation parameters of HPLC method for bearberry extract phytochemicals analysis

Analyte	Regression Equation	R^2	Tested linear range (µg/mL)	LOD (µg/mL)	LOQ (µg/mL)	Recovery (%)
Arbutin	y = 0.1421x + 0.1447	0.99998	1.5-200	1.150	3.484	100.76
Hydroquinone	y = 0.357x + 0.5011	0.99996	1.5-200	1.504	4.556	100.89
Picein	y = 0.7832x + 0.7798	0.99995	1.5-100	0.925	2.804	99.83
Methylarbutine	y = 0.1345x + 0.0073	0.99996	1.5-100	0.792	2.399	99.46
Corilagin	y = 0.5192x + 0.0657	0.99995	1.5-100	0.880	2.666	99.39
Pentagalloylglucose	y = 1.4537x + 0.6011	0.99994	1.5-100	1.023	3.099	101.15
Hyperoside	y = 0.801x + 0.7028	0.99993	1.5-100	1.079	3.270	101.38
Oleanolic acid	y = 0.2551x + 0.0815	0.99997	5-250	1.898	5.753	100.01
Ursolic acid	y = 0.1538x - 0.2692	0.99996	5-250	2.280	6.910	99.87

Limit of detection (LOD) and limit of quantification (LOQ) were calculated as 3.3σ/S and 10σ /S, respectively.
